# The role of L1 in L2 speech production at different stages of L2 development: Evidence from L2 Chinese oral production of verb-phrase ellipsis by English and Korean speakers

**DOI:** 10.3389/fpsyg.2022.954217

**Published:** 2022-11-29

**Authors:** Lulu Zhang, Boping Yuan

**Affiliations:** ^1^College of Foreign Languages and Literature, Fudan University, Shanghai, China; ^2^School of Foreign Languages, Shanghai Jiao Tong University, Shanghai, China; ^3^Faculty of Asian and Middle Eastern Studies, University of Cambridge, Cambridge, United Kingdom

**Keywords:** L2 Chinese, English- and Korean-speaking learners, oral production, L1 influence, verb-phrase ellipsis

## Abstract

The article reports on an empirical study investigating the role of L1 at the initial and developmental stages of L2 speech production. It examines two types of Chinese verb- phrase-ellipsis, ΣP-ellipsis licensed by the auxiliary *shi* ‘BE’ and vP-ellipsis licensed by the other auxiliaries, in 45 English and 45 Korean adult speakers’ L2 Chinese speech production. An elicited imitation task was administered to L2 learners at beginner, intermediate and advanced Chinese proficiency levels. L1 influence is not observed at beginner levels, but it appears at intermediate and advanced levels, L1 influence disappears at different time in English and Korean learners’ oral production of verb-ellipsis and ΣP-ellipsis. It is proposed that the absence of L1 influence at beginner levels is due to a breakdown of syntax-stylistics interface and beginners’ difficulty in implementing checking and deleting operations in their L2 oral production. The different timings of the disappearance of L1 influence in the two language groups at advanced levels is attributed to interactions between the persistence of L1 influence and the computational complexity involved in the target elliptical structures.

## Introduction

It is widely observed that in contrast to the uniform success of children acquiring their first language (L1), few adult learners can achieve native-like competence in their acquisition of a second language (L2). Obviously, L2 learners already have a language, i.e., their L1, in their mind, which can exert influence on their L2 acquisition. (FTFA, Schwartz and Sprouse, 1994, 1996) propose an influential model called Full Transfer (FT) Hypothesis^1^ in L2 acquisition research, which assumes that L1 grammar is transferred in its entirety to the initial state of L2 grammar. According to this hypothesis, the final state of grammatical properties of speakers’ L1 constitutes the initial state of their L2 grammars, and the development of L2 grammars is failure-driven; that is, when the L2 grammar is not able to accommodate data in the target language input, it is restructured on the basis of the input. This hypothesis has been supported by a substantial amount of evidence in L2 acquisition research (e.g., Hawkins, 2001; Haznedar, 2001; Slabakova, 2013) and few researchers would deny the fact that L1 does play a role in L2 acquisition. It is also well-documented that positive L1 transfer can facilitate L2 acquisition, and learners with L1 structures similar to or the same as those in the target language can acquire the target structures easier than those without (e.g., Inagaki, 2002; Slabakova, 2015; Zufferey et al., 2015). However, some researchers have also noticed that L1 influence is not inevitable, and it can be overridden in L2 acquisition (e.g., Montrul, 2010; Scheidnes and Tuller, 2010; Prévost et al., 2014). For instance, in Yuan (2015), which investigates the acquisition of attitude-bearing *daodi*…*wh-*questions in L1 English learners’ L2 Chinese, it is argued that L1 influence in L2 acquisition can be overridden by computational complexity. Specifically, unlike English *wh-*questions, where a *wh-*word is required to be raised from its base-generated position to the initial position of a sentence, a *wh-*word in Chinese *wh-*questions remains *in situ*. However, his study finds no evidence in the results that *wh-*movement in English is transferred into L1 English learners’ L2 Chinese *wh-*questions and causes problem in this aspect of their L2 Chinese grammars. Also, Chinese and English share the same restriction on attitude-bearing *wh-*questions, which regulates that a question cannot have more than one attitude. Yuan’s study shows that English speakers are unable to rule out ungrammatical Chinese *wh-*questions with two attitude features embedded in them, indicating that the similarities between English and Chinese in attitude-bearing *wh-*questions have very limited facilitation to L1 English learners’ handling of L2 Chinese *wh-*questions with more than one attitude feature. On the basis of Prévost et al. (2014) and Scheidnes and Tuller (2010), Yuan (2015) argues that L1 transfer is a relative phenomenon rather than an absolute phenomenon in L2 acquisition, and it can be overridden by the computational complexity involved in a construction.

The present study is an attempt to track the role of L1 in L2 speech production at different stages of L2 development. It aims to examine whether L1 grammar is transferred to L2 oral production at initial stages of L2 development, as predicted by the FT Hypothesis (FTFA, Schwartz and Sprouse, 1994, 1996), how L1 influence varies in the L2 development and whether L1 influence is subject to constraints such as computational complexity of a grammatical structure in the development of the L2. The study focuses on L1 English and L1 Korean learners’ L2 Chinese oral production of two types of verb-phrase ellipsis in an elicited oral production task. Chinese, English and Korean differ from each other in allowing certain types of verb-phrase ellipsis, which enables us to scrutinise the role that L1 plays in L1 English and L1 Korean learners’ L2 Chinese oral production.

The article is structured as follows. Section “Cross-linguistic differences of verb-phrase ellipsis in Chinese, Korean and English” discusses syntactic analyses of verb-phrase ellipsis in Chinese, English and Korean, and Section “Prior studies of L1 influence on L2 oral production” briefly reviews prior studies on the L2 production of Chinese elliptical structures and outlines the research questions. Section “Present study” introduces the methodology of the present study, and Section “Results” reports the scoring methods and results. The results are discussed in Sections “Discussion” and “Conclusion” contains our conclusions.

## Cross-linguistic differences of verb-phrase ellipsis in Chinese, Korean, and English

Chinese allows two types of verb-phrase ellipsis: a verb-phrase ellipsis licensed by the auxiliary *shi是* “BE”, as exemplified in (1), and a verb-phrase ellipsis licensed by auxiliaries other than *shi* “BE”, as illustrated in (2). As noted in Soh (2007), the scope of ellipsis licensed by *shi* ‘BE’ is larger than that licensed by the other auxiliaries like *hui 会* ‘will’. As shown in the contrast between (1) and (2), the elided constituent in the latter includes the verb phrase *likai yingguo* “leave the UK”, whereas that in the former includes the auxiliary *hui* ‘will’ as well as the verb phrase *likai yingguo* ‘leave the UK’. Also, as can be seen in the contrast between (3) and (4), when containing the negator *bu 不* ‘not’ in the antecedent clause, the scope of ellipsis licensed by *shi* ‘BE’, as shown in (3), includes the negator *bu* ‘not’, but that licensed by the auxiliary *hui* ‘will’ does not, as shown in (4).

1. 张三 会 离开 英国， 李四 也 是 会 离开 英国。
  Zhangsan hui likai yingguo, Lisi ye shi hui likai yingguo.
  Zhangsan will leave the UK Lisi also BE will leave the UK.
  ‘Zhangsan will leave the UK, and Lisi will (leave the UK) too.’

2. 张三 会 离开 英国， 李四 也 会 离开 英国。
  Zhangsan hui likai yingguo, Lisi ye hui likai yingguo.
  Zhangsan will leave the UK Lisi also will leave the UK.
  ‘Zhangsan will leave the UK, and Lisi will (leave the UK) too.’

3. 张三 不 会 离开 英国， 李四 也 是 不 会 离开 英国。
  Zhangsan bu. hui likai yingguo, Lisi ye shi bu hui likai yingguo.
  Zhangsan not will leave the UK Lisi also BE not will leave the UK.
  ‘Zhangsan will leave the UK, and Lisi will (leave the UK) too.’

4. 张三 不 会 离开 英国， 李四 也 不 会 离开 英国。
  Zhangsan bu. hui likai yingguo, Lisi ye bu hui likai yingguo.
  Zhangsan not will leave the UK Lisi also not will leave the UK.
  ‘Zhangsan will not leave the UK, and Lisi will not (leave the UK) either.’

**Figure 1:**
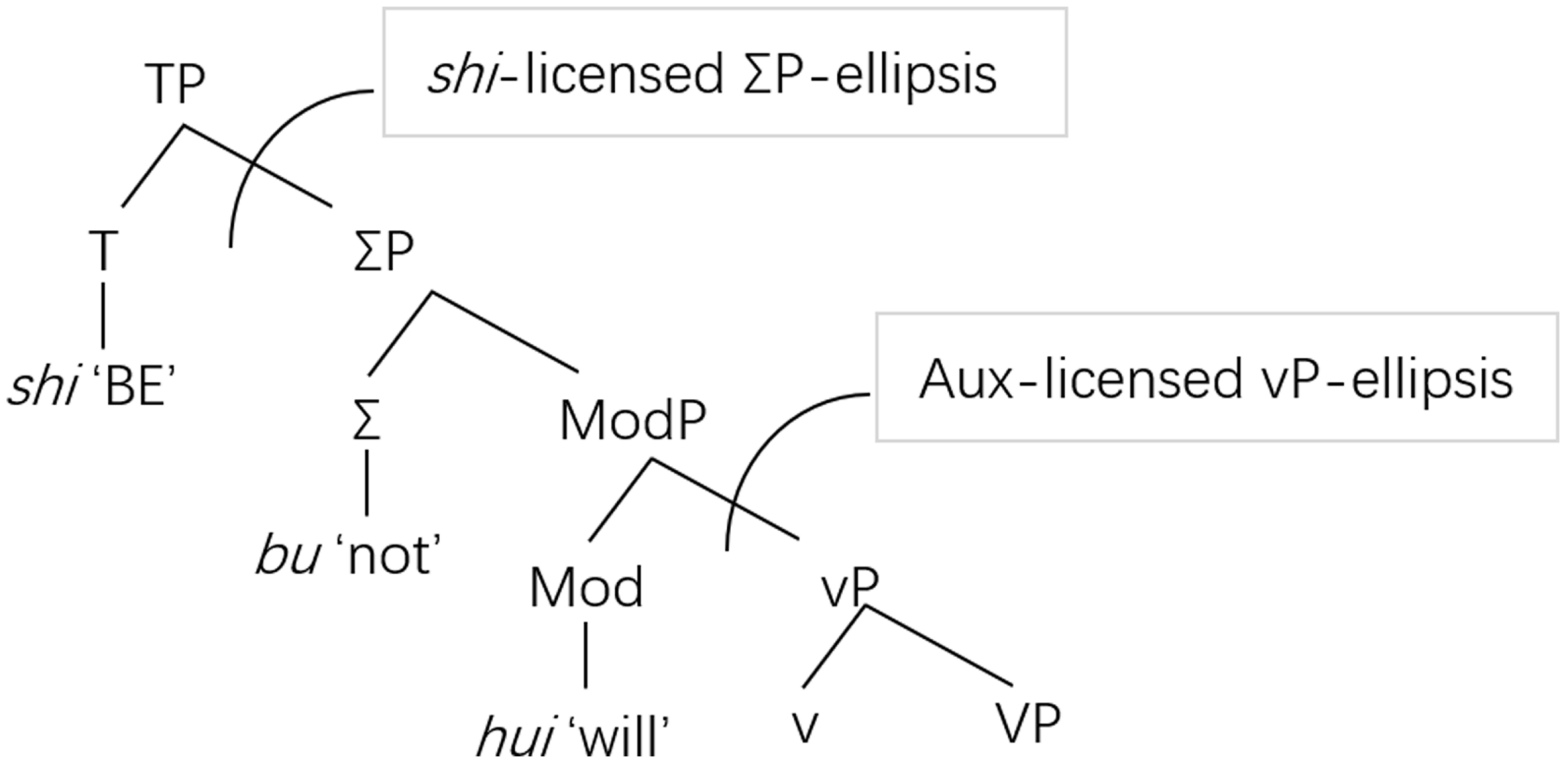
Positions of *shi* “BE” and auxiliaries in the hierarchy. Drawn using data from Soh (2007).

Based on the above observations, the present study follows Soh (2007) by assuming that *shi* ‘BE’, a dummy auxiliary in Chinese, occupies the head of TP, a position higher than the other auxiliaries such as *hui* ‘will’ in the hierarchy. Following Chomsky’s (1995) proposal that English auxiliaries are generated under Mod(al)P in the hierarchy, Soh (2007) argues that the auxiliaries in Chinese such as *hui* ‘will’ are generated under a Mod(al) node, which is lower than T, where *shi* ‘BE’ is located. The positions of the auxiliary *shi* ‘BE’ and the other auxiliaries in the hierarchy in Soh’s (2007) proposal are demonstrated in Figure 1. As can be seen, the auxiliaries exemplified by *hui* ‘will’ occupy the head of ModP, lower than the category Σ, which can be realized by the negator *bu* ‘not’ to express negative meaning; in contrast, *shi* ‘BE’ occupies the head of TP, which is higher than ΣP.

The fact that *shi* ‘BE’ and the other auxiliaries occupy different structural positions implies that they also differ in terms of the constituent they license. Thus, in line with Soh’s (2007) proposal, the scope of ellipsis licensed by *shi* ‘BE’ is a ΣP, whilst that licensed by the other auxiliaries like *hui* ‘will’ is a vP.

According to Li (2014), verb-phrase ellipsis in Chinese is a result of PF deletion. That is, when deriving a sentence with verb-phrase ellipsis, the elliptical verb phrase is first fully spelled out on the surface and then a deletion applies at the PF, resulting in the inaudibility of the verb phrase. A verb phrase can be deleted when verbal identity condition is met. That is, a vP or a ΣP can undergo deletion when it is identical to the antecedent in the first coordinate sentence (Chung, 2013; Liu, 2014; *cf.* Merchant, 2001).

It should be noted that complete sentences without verb-phrase ellipsis, as shown in (5), are perfectly grammatical in Chinese. However, they are stylistically heavy and unconcise, and thus are less preferred than the elliptical counterparts. Then, combining the above facts, we assume that in the derivation of a sentence with verb-phrase ellipsis, the deletion of the vP or ΣP is triggered at the syntax-stylistics interface, on the premise that the verbal identity condition is met.

5. 张三 会 离开 英国， 李四 也 会 离开 英国。
  Zhangsan hui likai yingguo, Lisi ye hui likai yingguo.
  Zhangsan will leave the UK Lisi also will leave the UK.
  ‘Zhangsan will leave the UK, and Lisi will leave the UK too.’

It is widely observed that English allows its verb-phrase ellipsis to be licensed by auxiliaries like *will* and *can* (Adger, 2003; *cf.* Johnson, 2001; Merchant, 2001, 2004). As shown in (6), sentences with ellipsis licensed by the auxiliary *will* in English seem to behave analogously to the Chinese vP-ellipsis licensed by auxiliaries in (2) and (4) respectively. Here we adopt Soh’s (2007) proposal that English auxiliaries are generated under a Modal node, occupying the head of ModP in the hierarchy. In this sense, like Chinese vP-ellipsis, what is elided in English elliptical sentences licensed by auxiliaries, like *will* in (6), is also a vP.

6. a. John will leave the UK and Bill will leave the UK too.
  b. John will not leave the UK, and Bill will not leave the UK either.

It is worth mentioning that a verb-phrase ellipsis in English can also be licensed by the dummy *do*, which is believed to behave similarly to the Chinese *shi* ‘BE’ in licensing some ΣP-ellipsis in English (Xu, 2003; Soh, 2007; Li and Wei, 2013). As can be seen in the Chinese sentence in (7) and its English translation, when the verb phrase *like Xiaohong* is elided, the dummy *do* is inserted to license the ellipsis in English, just like what *shi* ‘BE’ does in Chinese. However, unlike ΣP-ellipsis in Chinese, the scope of *do*-licensed verb-phrase ellipsis in English cannot include an auxiliary or negator, as exemplified in the sentences in (8). According to Soh (2007), this is because the English dummy *do* is positioned at the head of ModP, which is the same as the other auxiliaries in English but different from *shi* ‘BE’ in Chinese, which is at the head of TP. Consequently, what is elided after the dummy *do* is a vP rather than a ΣP, leading to the fact that English allows vP-ellipsis but not ΣP-ellipsis.

7. 张三 喜欢 小红， 李四 也 是 喜欢 小红
  Zhangsan xihuan Xiaohong, Lisi ye shi xihuan Xiaohong.
  Zhangsan like Xiaohong Lisi also BE like Xiaohong.
  ‘Zhangsan likes Xiaohong, and Lisi does (like Xiaohong) too.’

8. a. *Zhangsan will leave the UK, and Lisi does will leave the UK too.
  b. *Zhangsan will not leave the UK, and Lisi does will not leave the UK either.

It has been observed in the literature that languages like Japanese and Korean do not allow auxiliaries to license a verb-phrase ellipsis. This is because auxiliaries in these languages, like *-eul* ‘will’ in the Korean sentence in (9), are verbal suffixes, and thus deleting a verb phrase in a sentence and leaving the auxiliary alone would produce a ‘stray affix’^2^, rendering the remnant of the sentence ungrammatical.

9. 존이 영국을 떠날 거야. 빌도 영국을 떠날 거야.
  Jon-i yeonggug-eul tteona-l geoya. Bil-do yeonggug-eul tteona-l geoya.
  John-NOM England-ACC leave will Bill-too England-ACC leave will.
  ‘John will leave the UK, and Bill will (leave the UK) too.’

However, it is found that a verb-phrase ellipsis in Korean can be licensed by a lexical item *ya야* ‘BE’^3^ (Kim and Sohn, 1998). As illustrated in sentences in (10), the scope of the ellipsis licensed by *ya* ‘BE’ in Korean can include an auxiliary (e.g., *yongkuk-eul donal-koeaya* ‘will leave the UK’ in (10a)) and a negator (e.g., *yongkuk-eul an donal-koeaya* ‘will not leave the UK’ in (10b)). According to Kim and Sohn (1998), the lexical item *ya* is inserted to the head of TP after a focus-movement process and the deletion of a ModP. Based on the derivation analysis and the examples above, it can be summarised that the lexical item *ya* occupies the head of TP, a position higher than auxiliaries in Korean. On the basis of this analysis, we can assume that in Korean, the scope of the ellipsis licensed by *ya* is a ΣP, which can include an auxiliary and a negator, and that Korean allows ΣP-ellipsis, but not vP-ellipsis.

10. a. 존이 영국을 떠날 거야. 빌도 영국을 떠날 거야.

10. a. Jon-i yeonggug-eul tteona-l geoya. Bil-do yeonggug-eul tteona-l geoya.
  John-NOM England-ACC leave-will Bill-too England-ACC leave-will.
  ‘John will leave the UK, and Bill will (leave the UK) too.’

b. 존이 영국을 떠나지 않을 거야. 빌도 영국을.
  Jon-i yeonggug-eul tteona-ji an-heul geoya. Bil-do yeonggug-eul.
  John-NOM England-ACC leave not will Bill-too England-ACC.
  떠나지 않을 거야.
  tteona-ji an-heul geoya.
  leave not will.
  ‘John will not leave the UK, and Bill will not (leave the UK) either.’

Cross-linguistic differences with regard to the availability of vP-ellipsis and ΣP-ellipsis are summarised in Table 1.

**Table 1:** Summary of the availability of vP-ellipsis and ΣP-ellipsis in Chinese, English and Korean.

	Chinese	English	Korean
vP-ellipsis	+ (Aux-licensed)	+ (Aux-licensed & *do*-licensed)	–
ΣP-ellipsis	+ (*shi*-licensed)	–	+ (*ya*-licensed)

## Prior studies of L1 influence on L2 oral production

Much evidence of L1 influence on adult L2 speech production has been reported in the literature, and many features in L2 oral production find their origin in the.

L1 (see overviews in Kellerman and Sharwood Smith, 1986; Gass and Selinker, 1992; Odlin, 1989, 2003). However, evidence has been emerging in the literature that L2 influence in L2 speech production is not inevitable, and it is argued in 2008Yuan (2001) that L1 influence is not everywhere. His argument is based on oral production data and judgment data concerning thematic-verb raising collected from adult French-, German- and English-speaking learners of L2 Chinese. Thematic verbs are allowed to raise in French and German, but not in English and Chinese, and Yuan’s findings show that neither French- nor German-speaking adult learners’ L2 oral production of Chinese is influenced by the thematic-verb raising in their L1 French and German, which shows clear absence of L1 transfer in L2 speech production and provides evidence against the FT Hypothesis (FTFA, Schwartz and Sprouse, 1994, 1996).

Absence of L1 influence is also reported in Hawkins and Casillas (2008), although their study is not to confirm or disconfirm the FT Hypothesis. In their study, adult Chinese- and Spanish-speaking learners of English are examined for their use of subject-verb agreement in their L2 English. Subject-verb agreement is realised in.

Spanish but not in Chinese, and if properties of L1 verb morphology are influential in the acquisition of English, this difference should show up in the performance of the.

two groups. Results of an oral completion task show that both groups perform strikingly similarly; (i) the copula */is/* is supplied more than the 3^rd^ person singular */s/* with simple subjects, and there is no overgeneralisation of */is/* or */s/* when the subject is plural; (ii) there is no decrease in the suppliance of */is/* or */s/* when there is a complex subject; (iii) suppliance of */s/* with a complex subject is disrupted when there is an intervening prepositional phrase (PP); however, suppliance of the copula */is/* is only disrupted where the PP contains a plural N, not when both Ns are singular. The similar behaviours of the two groups in Hawkins and Casillas (2008) suggest again that the L1 is unlikely to be influential in determining the knowledge that gives rise to these patterns of behaviours.

While evidence for the absence of L1 influence in L2 speech production is emerging, it is still not as robust as evidence for such influence. In addition, the variety of language phenomena tested for the former is still rather limited. More importantly, answers are yet to be found as to why there is absence of L1 influence on some L2 structures given that L1 transfer is a rather pervasive phenomenon in L2 speech production.

There has been considerable linguistic research on the syntactic mechanism underlying ellipsis (e.g., Grinder and Postal, 1971; Lobeck, 1995; Kehler, 2000; Johnson, 2001; Hendriks, 2004; Kertz, 2013), as well as psycholinguistic research examining parallelism effects on ellipsis (Arregui et al., 2006; Matsuo, 2007; Frazier, 2008; e.g., Matsuo and Duffield, 2001), but only a few studies have investigated elliptical structures in L2 speech production, one of which is Yuan and Zhang’s (2020) study, which investigates object ellipsis in L2 Chinese speech production by adult L1 Korean and L1 English learners at various L2 Chinese proficiency levels. They adopt an analysis of Chinese object-ellipsis structures on the basis of topicalization and topic deletion (Li and Thompson, 1981; Huang, 1984), and argue that the equivalent of object ellipsis is allowed in Korean but not in English. An elicited imitation task^4^ was used to test L2 speech production of the target elliptical structures. In the study, both Korean- and English-speaking beginner learners of L2 Chinese are found to overwhelmingly produce utterances with overt objects after they hear sentences with object ellipsis. The authors’ explanation for the absence of the object ellipsis in L2 Chinese beginners’ oral production is based on an incremental model for speech production (adapted from Bock and Levelt, 1994). The model proposes four stages during the grammatical encoding for speech production. Specifically, lexical concepts and lemmas are selected for conveying the message at the first stage, and are assigned grammatical functions at the second stage. At the third stage, the constituents are assembled in a word order suitable for the target sentence, while at the final derivation stage some procedures such as movement and deletion take place before the sentence is spelt out. The authors argue that beginner learners encounter problems in handling the movement and deletion procedures at the derivation stage, rendering the overwhelming production of non-ellipsis responses in L1 English and L1 Korean beginners’ L2 Chinese oral production. Another finding of the study is that results of both the elicited imitation task and an acceptability judgment task suggest no L1 influence on speech production throughout L2 Chinese developmental stages, as no significant difference is found between L1 English groups and proficiency-matched L1 Korean groups. However, no specific account is provided in Yuan and Zhang (2020) as to why no L1 influence is found in their study. Another study reported in Zhang (2020) yields an inconsistent finding. This study explores the role of L1 in L2 acquisition of verb-phrase ellipsis, and the results are discussed from the perspective of structural priming effect, i.e., whether language users tend to reuse the same grammatical structure as the one in recent discourse (Bock, 1986). Specifically, Zhang (2020) examines data from an elicited imitation task by 77 intermediate L1 English and L1 Korean learners of L2 Chinese. The data shows an obvious difference between L1 English and L1 Korean groups; when primed for a certain type of verb-phrase ellipsis structure, learners whose L1 has the equivalent of the ellipsis type produce significantly more responses with the primed ellipsis structure, displaying a significantly stronger priming effect than those whose L1 does not have the equivalent. The author attributes the between-group difference to L1 influence, and concludes that at intermediate levels, learners’ L2 speech production is affected by the presence or absence of the equivalent of the primed structure in their L1s. This finding supports the language-nonspecific account in Flett et al. (2013), which argues that the magnitude of a structure’s priming effect in L2 speech production is influenced by both the speaker’s L2 and L1, rather than by their L2 only. The finding, however, left a question unanswered as to why L1 difference is found in L2 Chinese production of verb-phrase ellipsis, but is absent in L2 Chinese production of object ellipsis, as observed by Yuan and Zhang (2020). More importantly, since Zhang (2020) focuses on intermediate learners of L2 Chinese in her study, it remains unclear whether the significant difference between different L1 groups’ L2 Chinese oral production of verb-phrase ellipsis occurs at stages before the intermediate level, particularly at beginner levels, and whether it can be overcome beyond the intermediate level. Thus, the unexplored questions become the aims of the present study, which is to provide a full picture about the role of L1 in L2 Chinese speech production during the L2 development, from beginner to advanced levels. Attempts are to be made to account for the occurrence and disappearance of L1 influence in L2 Chinese oral production of vP- and ΣP-ellipsis.

## Present study

### Research questions and predictions

On the basis of the cross-linguistic differences with regard to the (un)availability of vP-ellipsis and ΣP-ellipsis in English, Korean as well as Chinese, the following research questions are asked in this study.

*Research Question 1.* Is English- and Korean-speaking L2 Chinese beginners’ oral production of vP- and ΣP-ellipses influenced by their L1s?

*Predictions:* On the basis of the Full Transfer (FT) Hypothesis (FTFA, Schwartz and Sprouse, 1994, 1996) and given the fact that English allows vP-ellipsis but not ΣP-ellipsis while Korean allows the latter but not the former, it is predicted that L1 influence will occur in beginners’ L2 Chinese speech production and that the L1 influence will lead to differences between English and Korean in (dis)allowing vP-ellipsis and ΣP-ellipsis at L2 Chinese beginner levels. Specifically, (i) L1 English beginners are predicted to produce more vP-ellipsis sentences in their L2 Chinese speech production than L1 Korean beginners; (ii) L1 Korean beginners are predicted to produce more ΣP-ellipsis sentences in their L2 Chinese speech production than L1 English beginners.

*Research Question 2.* To what extent does L1 play a role in L2 Chinese oral production of vP- and ΣP-ellipsis at different stages of the L2 Chinese development? Does L1 influence persist or disappear at advanced levels? Specifically, do L1 English and L1 Korean advanced learners of L2 Chinese behave similarly in their oral production of sentences with vP- or ΣP-ellipsis?

*Predictions:* If L1 influence persists at the advanced level, L1 English and L1 Korean advanced learners will behave differently to each other in their oral production of target sentences with vP- or ΣP-ellipsis; if L1 influence can be overcome, advanced learners from different L1 backgrounds will not differ significantly in their oral production of target sentences.

### Participants

The total number of participants in the empirical study is 105, which includes 45 adult L1 English and 45 adult L1 Korean learners of L2 Chinese as well as 15 adult native Chinese speakers as a control group. They were mainly students from universities in Britain and China at the time of data collection. The L1 English and L1 Korean participants all had previously received classroom instruction in Chinese language, and most of them had spent a certain period of time in China by the time of the experiment. Native English speakers who had learned any East Asian languages other than Chinese, such as Korean or Japanese, were excluded. For native Korean speakers, as English is a compulsory course in universities in South Korea, it is unavoidable that all of them have learned English for some time. Those who had not been to any English-speaking country and self-rated their English as lower than advanced level (i.e., elementary level or intermediate level) were selected. Payments were given to every participant as a token of thanks for their participation in the study.

Participants’ working memory capacity is also controlled. The task chosen in the current study to test participants’ working memory capacity is the backward digit span task, which is one of the subtests of Wechsler Adult Intelligence Scale–Fourth UK Edition (Wechsler, 2010), and has been used in recent literature (Gathercole et al., 2004, 2008; Gathercole and Alloway, 2007; Hsieh, 2015). In the task, participants first listen to a digit span (one digit per second) read in their native languages and then are required to repeat the span backwards. The score of the task is the highest number of digits that a participant is able to correctly repeat. To ensure that participants have similar working memory capacity, those who are only able to correctly repeat fewer than 6 digits are excluded from the study. The statistical data and the results of one-way ANOVA of the backward digit span test scores of different L1 groups are shown in Table 2.

**Table 2:** Results of the backward digit span test.

English speakers		Korean speakers		Chinese speakers			
Mean	*SD*	Mean	*SD*	Mean	*SD*	*F* (2,102)	*p*
6.71	0.46	6.62	0.49	6.53	0.52	0.880	0.418

The remaining participants are divided into seven groups based on their native languages and their performance in a cloze test. The cloze test is adopted from Mai and Yuan (2016), which consists of 3 passages and contains 40 gaps in total. Participants are required to fill in the gaps using correct Chinese characters or Pinyin (an alphabetical system for Chinese pronunciation). The maximum number of correct responses in this test is 40. Information of the participants and results of the cloze test for each group are given in Table 3. A one-way ANOVA is administrated on the cloze test scores between the learner groups and the NS Group, and the results reveal a significant difference between the groups in their performance in the cloze test (*F*(6, 98) = 465.763, *p* < 0.001). *Post hoc* Scheffé tests indicate that all learner groups are significantly different from the NS group. The results also show that there is no significant difference between any of the two corresponding language groups in their scores in the cloze test; that is, no significant difference is found between the EB Group and the KB Group (*p* > 0.05), between the EI Group and the KI Group (*p* > 0.05), or between the EA Group and the KA Group (*p* > 0.05). These indicate that all of the English groups are compatible with their corresponding Korean groups with regard to their Chinese language proficiency.

**Table 3:** Information about participants in each group.

Groups	n (male/ female)	Age		Onset age of learning Chinese		Time spent learning Chinese (months)		Duration of stay in China (months)		Cloze test	
		Mean (range)	*SD*	Mean (range)	*SD*	Mean (range)	*SD*	Mean (range)	*SD*	Mean (range)	*SD*
EB	15 (8/7)	22 (17–27)	3.23	20 (17–25)	2.76	17 (4–48)	14.65	2 (0–16)	5.40	6 (6–13)	1.86
EI	15 (8/7)	21 (19–27)	2.27	18 (17–25)	2.05	34 (5–96)	31.08	7 (0–15)	5.97	19 (15–24)	2.88
EA	15 (7/8)	23 (21–29)	2.72	17 (15–22)	1.63	58 (38–108)	17.82	17 (10–30)	6.67	33 (30–37)	2.43
KB	15 (7/8)	22 (18–25)	2.35	21 (17–25)	2.58	7 (1–24)	6.83	2 (1–8)	2.13	6 (6–13)	2.06
KI	15 (8/7)	22 (19–25)	1.99	19 (17–23)	1.76	36 (3–84)	25.07	27 (1–72)	20.92	19 (16–24)	2.59
KA	15 (6/9)	22 (18–28)	2.53	17 (15–20)	1.41	61 (36–96)	22.47	47 (6–72)	18.19	32 (29–37)	2.37
NS	15 (9/6)	24 (18–30)	3.75	N/A	N/A	N/A	N/A	N/A	N/A	39 (36–40)	1.28

EB, English Beginner Group; EI, English Intermediate Group; EA, English Advanced Group; KB, Korean Beginner Group; KI, Korean Intermediate Group; KA, Korean Advanced Group; NS, Native Speaker Group.

### Instruments

Participants are required to complete a language background questionnaire and the working memory test prior to the main experiment, which includes an elicited imitation task^5^ for eliciting L2 learners’ Chinese speech production of sentences with vP- or ΣP-ellipsis.

Before the experiment begins, each participant is required to read aloud the words and phrases on a vocabulary list for the experiment and tell the administrator the meaning of each character/phrase. This is to make sure that their performance in the task is not to be affected by vocabulary issues. Both written and oral instructions are provided in participants’ L1s, and five practice trials are given to the participant before the experiment starts. In the experiment, recorded utterances are presented to the participant auditorily one by one, and then the participant is prompted to recall the utterance orally. On each trial in the experiment, participants first read contextual information conveyed by a picture on the computer screen and a sentence or phrases under or around the picture, and then click a speaker icon on the upper left corner of the screen to listen to an audio file that contains the eliciting utterance. Each eliciting utterance is preceded by a chiming sound to alert participants to listen. After the audio presentation of the eliciting utterance, the participant would hear an instruction in Chinese *qing huida* ‘please answer’. Participants are then required to make a decision about whether the sentence they have just heard matches the contextual information on the screen, by selecting an option of “Match,” “Mismatch” or “I do not know” on an answer sheet provided. This serves as a comprehension task to draw participants’ attention to the meaning rather than the form of the eliciting utterance. This also provides a way to measure participants’ comprehension of the utterance. Obviously, without correct comprehension, it would be difficult for the participant to recall the utterance. These procedures are also to ensure that there will be a time interval of at least 3 s between the presentation of the eliciting utterance and the start of the recalling. All this helps to make sure that the utterance produced by the participant is reconstructive, “requiring participants to process, rather than repeat verbatim, language stimuli” (Erlam, 2009, p. 488). Participants are then required to orally recall the utterance they have heard in Chinese immediately, which is to force participants to perform the recalling with time pressure instead of being self-paced, and to ensure that participants have little time to plan or monitor their responses.

This design is adapted from the methods used in Erlam’s (2006, 2009) and Chrabaszcz and Jiang’s (2014) studies. The rationale behind the elicited imitation task is the requirement for a participant to “decode the sentence they hear through syntactic and semantic parsing, retain the meaning, and reconstruct the sentence for subsequent production” (Chrabaszcz and Jiang, 2014, p. 359).

### Materials

**Figure 2:**
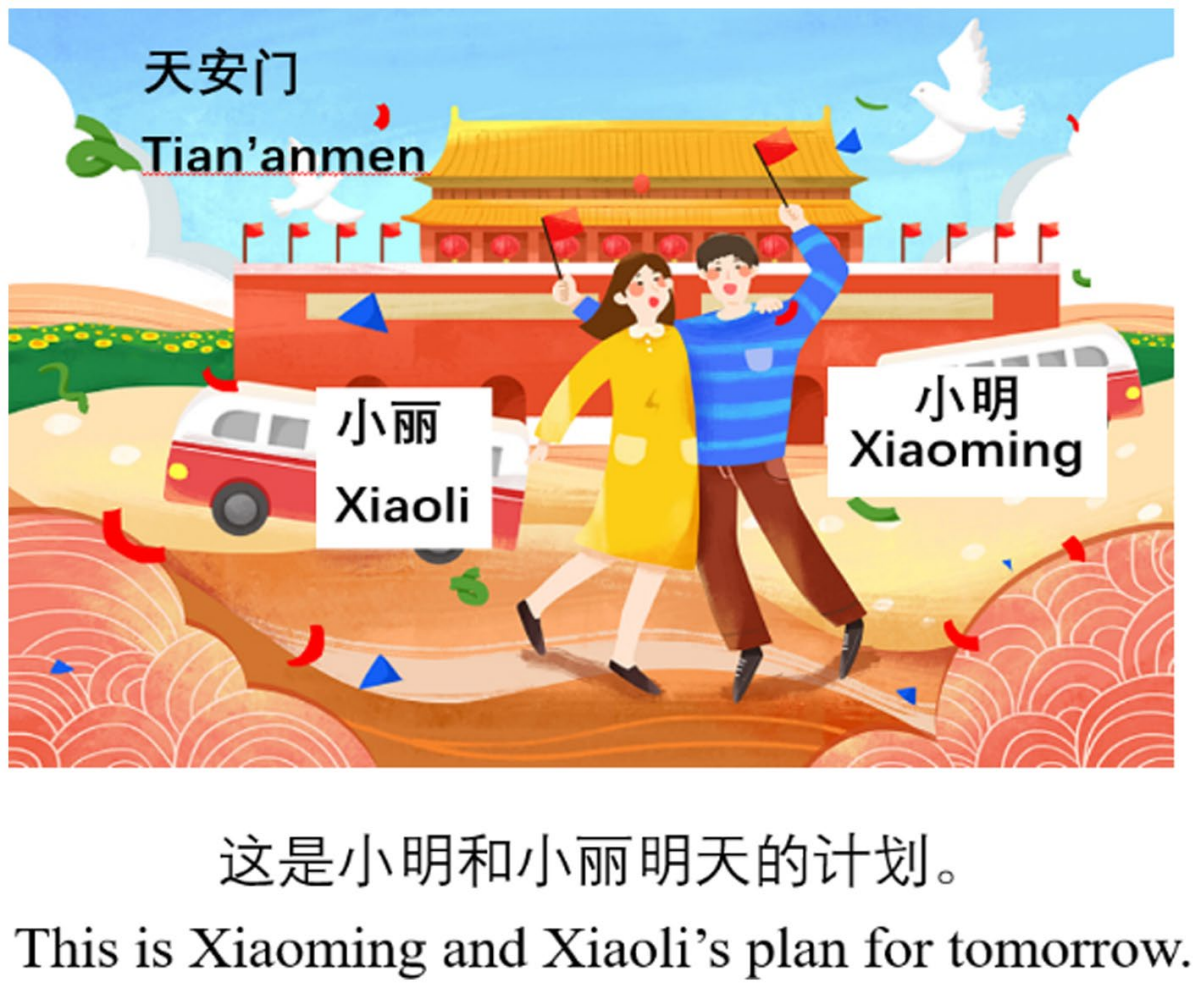
Picture and contextual information for (11).

**Figure 3:**
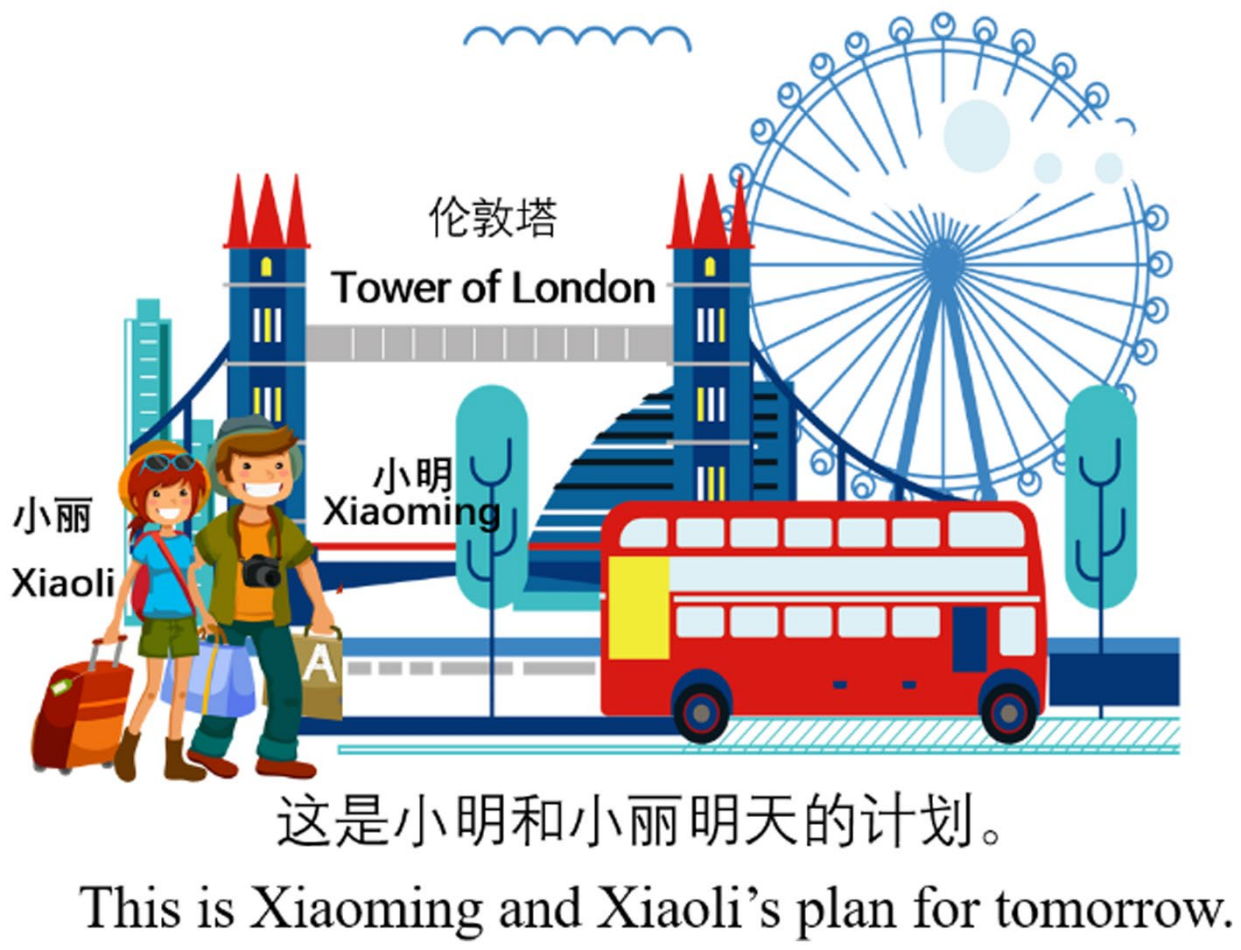
Picture and contextual information for (12).

There are 70 sentences in audio files during the experiment, out of which 12 are related to the investigation of L2 Chinese vP-ellipsis and ΣP-ellipsis, whilst the rest serve as distracters in the experiment. The 12 sentences are in two conditions, i.e., vP- and ΣP-ellipsis conditions (as illustrated in (11) and (12)), with each condition having six test sentences. Each test sentence consists of three clauses, and each sentence contains 22 or 23 Chinese characters. The pictures and contextual information for (11) and (12) are provided in Figures 2, 3 respectively. In the experiment, the contextual information only uses Chinese characters, and the English translation in Figures 2, 3 is provided for readers of this article.

11. Example of the vP-ellipsis condition:
  Mingtian Xiaoming hui qu Beijing, Xiaoli ye **hui**, tamen hui yiqi qu.
  Tomorrow Xiaoming will go Beijing Xiaoli also will they will together go.
  kan Tian’an Men.
  see Tian’an Men.
  ‘Xiaoming will go to Beijing tomorrow, and Xiaoli will too. They will go to see Tian’an Men together.’

12. Example of the ΣP-ellipsis condition:
  Mingtian Xiaoming hui qu Lundun, Xiaoli ye shi. Tamen dou shi diyi-ci.
  tomorrow Xiaoming will go London Xiaoli also BE they both be first-CL.
  chuguo lvxing.
  go abroad travel.
  ‘Xiaoming will go to London tomorrow, and Xiaoli will too. This is their first time to travel abroad.’

### Categorizing and scoring participants’ responses

Participants’ responses in the elicited imitation task were recorded, transcribed and analysed. In the data trimming process, unclear responses, responses not immediately produced, and responses where the second clause is not produced or largely incomplete were removed and treated as missing values. Note that the choice of incorrect names (e.g., *Xiao Wei* instead of *Xiao Li*) and replacement of verbs or nouns with synonyms, were not treated as incorrect responses, as this kind of mistake does not affect the use of ellipsis. We categorized the responses into four categories, as described in (13).

13. Categorization of responses:

- Responses with vP-ellipsis, where the second clause of the sentence does not have a phonetically realized verb phrase following an auxiliary;
- Responses with ΣP-ellipsis, where the second clause of the sentence does not have a phonetically realized model verb phrase following *shi* ‘BE’;
- Responses with object ellipsis, where the second clause of the sentence has a transitive verb, but does not have a phonetically realized object;
- Responses with no ellipsis, where no vP, or ΣP or object in the second clause is elided.

Next, we gave 1 to each response representing one of the categories described above. The score and percentage of each response category were calculated for each participant group, respectively. Recall that six test sentences are contained in each condition, and each group contains 15 participants. A group’s maximum the accumulated score for a response category in a condition is 90 and the minimum is 0.

## Results

### Results of the comprehension test

Recall that a comprehension test is included in the elicited imitation task, which requires participants to choose an option of “Match,” “Mismatch” or “I do not know” on the answer sheet. Their comprehension of the sentences is checked by analysing the accuracy of their Match/Mismatch choices. Participants who correctly judged more than 63 items out of 70 (i.e., accuracy above 90%) were considered to have attended to meanings of the eliciting sentences. The results show that all groups’ accuracy rates are higher than 93%, indicating that they have good comprehensions of the eliciting sentences in the task.

### Data analysis

#### Native speakers of Chinese

As can be seen in Table 4, in both vP-ellipsis and ΣP-ellipsis conditions, native Chinese speakers produce an overwhelming number of target structures in their responses. Specifically, their vP-ellipsis responses account for 79% of the responses in the vP-ellipsis condition and ΣP-ellipsis responses 69% of the responses in the ΣP-ellipsis condition, whist non-target responses in both conditions are all under 15%. As the present study focuses on the L1 influence on English and Korean speakers’ oral production of sentences with vP or ΣP-ellipsis, the native speakers’ data in the study only serves as the baseline for response choices and will not be discussed further.

**Table 4:** The number of each response category in vP-ellipsis and ΣP-ellipsis conditions across groups (percentages in parentheses).

	vP-ellipsis Condition				ΣP-ellipsis Condition			
	Target	Non-Target			Target	Non-Target		
	vP-E	Non-E	Object-E	ΣP-E	ΣP-E	Non-E	Object-E	vP-E
EB	7 (8%)	79 (88%)	4 (4%)	0 (0%)	0 (0%)	81 (90%)	6 (7%)	3 (3%)
EI	40 (42%)	39 (41%)	17 (18%)	0 (0%)	6 (6%)	42 (44%)	23 (24%)	25 (26%)
EA	50 (60%)	12 (14%)	20 (24%)	2 (2%)	14 (17%)	14 (17%)	23 (28%)	32 (38%)
KB	2 (2%)	78 (93%)	4 (5%)	0 (0%)	1 (1%)	81 (96%)	2 (2%)	0 (0%)
KI	14 (15%)	46 (48%)	14 (15%)	22 (23%)	39 (41%)	47 (49%)	8 (8%)	2 (2%)
KA	39 (43%)	19 (21%)	15 (17%)	17 (19%)	45 (50%)	17 (19%)	13 (14%)	15 (17%)
NS	71 (79%)	7 (8%)	10 (11%)	2 (2%)	62 (69%)	7 (8%)	8 (9%)	13 (14%)

EB, English Beginner Group; EI, English Intermediate Group; EA, English Advanced Group; KB, Korean Beginner Group; KI, Korean Intermediate Group; KA, Korean Advanced Group; NS, Native Speaker Group; vP-E, responses with vP-ellipsis; Non-E, responses with no ellipsis; Object-E, responses with object ellipsis; ΣP-E, responses with ΣP-ellipsis.

#### L2 groups

As shown in Table 4, great variations can be found in L2 groups’ responses. In the vP-ellipsis condition, the EB Group behave similarly to the KB Group, producing very few target responses (the EB Group: 8% and the KB Group: 2%), even though vP-ellipsis is allowed in English; instead, both beginner groups produce a large proportion of responses with overt vP (the EB Group: 88% and the KB Group: 93%) in spite of the fact that no overt vP is included in the eliciting utterance. This provides us with evidence that no L1 transfer takes place at beginning levels of L2 Chinese oral production of vP-ellipsis. As their Chinese proficiency improves, the L2 learners produce increasingly more target responses with vP-ellipsis (the EI Group: 42%, the EA Group: 60%, the KI Group:15%, and the KA Group: 43%); at the same time, the frequencies of responses with non-ellipsis dramatically decrease (the EI Group: 41%, the EA Group: 14%, the KI Group: 48%, and the KA Group: 21%).

In the ΣP-ellipsis condition, the two beginner groups again behave similarly; they rarely produce target responses with ΣP-ellipsis (the EB Group: 0% and the KB Group: 1%) even though ΣP-ellipsis is allowed in Korean. In contrast, they produce responses with overt ΣP at very high rates (the EB Group: 90% and the KB Group: 96%), in spite of that fact that the eliciting utterances contain ΣP-ellipsis. Again, absence of L1 transfer is observed in beginners’ oral production of ΣP-ellipsis. With the increase of their Chinese proficiency at intermediate and advanced levels, L1 Korean groups produce an increasingly higher proportions of target responses with ΣP-ellipsis (the KI Group: 41%, and the KA Group: 50%) than L1 English groups (the EI Group: 6%, and the EA Group: 17%).

The number of L2 learners’ target responses were submitted to a linear mixed-effect models under the lme4 package in R version 4.1.0 (R Development Core Team, 2021). The fixed predictors include Proficiency (categorical factor, sum coded: beginner = −1, intermediate = 0, and advanced = 1), L1 (categorical factor, sum coded: English = −1 and Korean = 1) and Condition (categorical factor, sum coded: vP-ellipsis = −1 and ΣP-ellipsis = 1), and the interactions of Proficiency * L1, Proficiency * Condition, Condition * L1, and Condition * L1 * Proficiency. Participant and test items were entered as random factors for intercepts and slopes. A maximal model was first established, based on which the optimal model was found by backword elimination procedure. The formula of the optimal model is Score ~ Condition x L1 x Proficiency + (1 + L1 + Condition | Participant) + (1 + Proficiency + L1 | Item).

**Figure 4:**
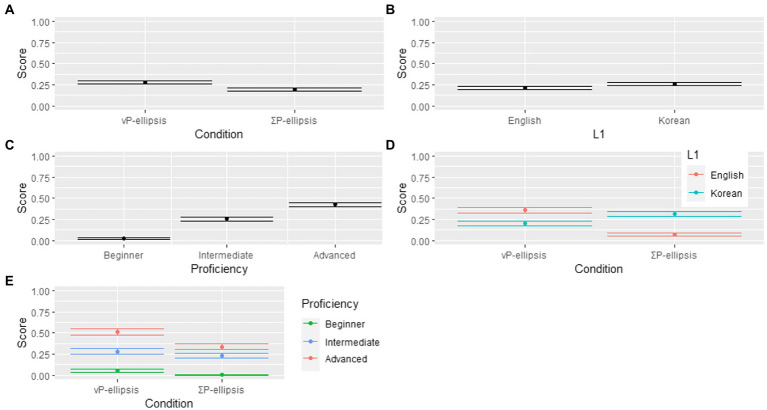
Score as a function of Condition, Proficiency, and L1. **(A)** Score as a function of Condition. **(B)** Score as a function of L1. **(C)** Score as a function of Proficiency. **(D)** Score as a function of Condition by L1. **(E)** Score as a function of Condition by Proficiency.

The model output is presented in Table 5 and Figure 4. The results reveal a significant three-way Condition * L1 * Proficiency interaction, which indicates that the interaction of Condition and L1 differed across three proficiency levels. Specifically, from Figure 4A, it can be observed that the score is very close between ΣP-ellipsis condition and vP-ellipsis condition, although the score is slightly higher for vP-ellipsis condition than ΣP-ellipsis condition. The statistical data in Table 5 confirm that the effects of Condition is non-significant (*p* = 0.149). Similarly, Figure 4B shows that the score of English learners of Chinese is only slightly lower than that of Korean learners of Chinese, and the data in Table 5 reveal that the effect of L1 is non-significant (*p* = 0.444). In contrast, Figure 4D shows that the difference in the score between English and Korean learners is clearly different in ΣP-ellipsis condition than it is in vP-ellipsis condition (one difference is positive, the other negative), and this significant difference is confirmed by the statistical result of interaction between Condition and L1 (*p* **<** 0.001). Consequently, there is no overall effect of either L1 or condition, but there is a crossover interaction. From Figure 4C, it can be observed that the score is proportionate with proficiency, and the statistical data in Table 5 reveal that the effect of Proficiency is significant (*p* **<** 0.001). This indicates that the number of target responses varied across different proficiency groups. Figure 4E shows that the effect of proficiency is similar in ΣP-ellipsis condition and vP-ellipsis condition. This echoed the patten in Figure 4E that the difference among three proficiency groups’ scores across the two conditions are very similar, and thus the effect of Condition and Proficiency is not significant (*p* = 0.279).

**Table 5:** Summary of the linear mixed-effect models for target responses.

	Score				
Predictors	Estimates	std. Error	CI	Statistic	*p*
(Intercept)	0.24	0.03	0.18–0.30	7.92	<0.001
Condition	−0.09	0.06	−0.21 – 0.03	−1.44	0.149
L1	0.03	0.04	−0.05 – 0.12	0.77	0.444
Proficiency	0.40	0.06	0.27–0.52	6.39	<0.001
Condition * L1	0.40	0.09	0.22–0.58	4.31	<0.001
Condition * Proficiency	−0.14	0.13	−0.39 – 0.11	−1.08	0.279
L1 * Proficiency	0.11	0.07	−0.03 – 0.24	1.52	0.128
Condition * L1 * Proficiency	0.43	0.16	0.12–0.73	2.75	0.006
Observations	1,080				

In order to explore difference between target responses of proficiency-matched L1 English and L1 Korean groups, thereby answering the questions about the occurrence and disappearance of L1 influence at different L2 stages, Turkey *post hoc* comparisons were conducted through the emmeans package (Lenth, 2020) in a simplified model using dummy coding (formula: Score ~ Condition × L1 × Proficiency + (1 + L1 | Participant) + (1 + Proficiency + L1 | Item)). To explore the answer to the first research question about the role of L1 in L2 Chinese beginners’ oral production, beginner learner groups’ results were examined. The results suggest that there is no L1-related difference between L1 English and L1 Korean beginner learners’ L2 Chinese production of utterances with vP- and ΣP-ellipsis (vP-ellipsis condition, EB vs. KB: *β^* = 0.0518, *SE* = 0.0797, *t* = 0.649, *p* > 0.05; ΣP-ellipsis condition, EB vs. KB: *β^* = −0.0098, *SE* = 0.0798, *t* = −0.123, *p* > 0.05). This suggests that L1 English beginners do not produce more vP-ellipsis sentences in their L2 Chinese speech production than L1 Korean beginners, and L1 Korean beginners do not produce more ΣP-ellipsis sentences in their L2 Chinese speech production than L1 English beginners.

To explore the answer to the second research question about the role of L1 in L2 developmental stages, intermediate and advanced learner groups’ results were examined. In contrast, L1 difference is found to be a significant factor in L1 English and L1 Korean intermediate learners’ L2 Chinese production of vP- and ΣP-ellipsis (vP-ellipsis condition, EI vs. KI: *β^* = 0.2692, *SE* = 0.0779, *t* = 3.458, *p* < 0.05; ΣP-ellipsis condition, EI vs. KI: *β^* = −0.3421, *SE* = 0.0779, *t* = −4.394, *p* < 0.01), indicating that L1 influence is absent at the beginner levels, but occurs at the intermediate level. At the advanced level, L1 English and L1 Korean groups’ frequencies of target responses in the ΣP-ellipsis condition differ significantly from each other (EA vs. KA: *β^* = −0.3318, *SE* = 0.0801, *t* = 4.141, *p* < 0.01), indicating the persistence of L1 influence in the two advanced groups’ L2 Chinese oral production of ΣP-ellipsis. In contrast, the advanced groups’ frequencies of target responses in the vP-ellipsis condition are not significantly different (EA vs. KA: *β^* = 0.1603, *SE* = 0.0801, *t* = 2.001, *p* > 0.05), suggesting disappearance of L1 influence in the two advanced groups’ L2 Chinese oral production of vP-ellipsis.

## Discussion

As the present study aims to investigate the role of L1 in L2 speech production of vP- and ΣP-ellipsis at different stages of L2 Chinese development, this section is to discuss findings of the investigation with answers to the research questions concerning the role of L1 in different developmental stages of L2 Chinese vP- and ΣP-ellipsis.

### Answers to research questions

*Research Question 1.* Is English- and Korean-speaking L2 Chinese beginners’ oral production of vP- and ΣP-ellipses influenced by their L1s?

The results suggest that L1 influence is absent at beginner levels, which provides us with evidence against the FT Hypothesis (FTFA, Schwartz and Sprouse, 1994, 1996), which argues that the L2 initial state is entirely based on the final state of learners’ L1. In our study, both L1 English and L1 Korean beginner learners of Chinese produce few utterances with vP- or ΣP-ellipsis even though the former is allowed in English and the latter in Korean; instead, they have overwhelming production of utterances with overt vP and ΣP in spite of the fact that the eliciting sentences contain vP- and ΣP-ellipsis. In contrast, L1 influence is found in the two intermediate groups’ L2 Chinese production of utterances with vP- and ΣP-ellipsis.

*Research Question 2.* To what extent does L1 play a role in L2 Chinese oral production of vP- and ΣP-ellipsis at different stages of the L2 Chinese development? Does L1 influence persist or disappear at advanced levels? Specifically, do L1 English and L1 Korean advanced learners of L2 Chinese behave similarly in their oral production of sentences with vP- or ΣP-ellipsis?

The results indicate an asymmetry in the persistence of L1 influence with regard to vP-ellipsis and ΣP-ellipsis at advanced learners’ L2 Chinese oral production. Specifically, the results reveal that at advanced levels, the difference between the L1 English and L1 Korean learners in producing utterances with vP-ellipsis disappears, as no significant difference is found between the frequencies of vP-ellipsis utterances in the EA and KA Groups. This is in contrast to the ΣP-ellipsis condition, where the EA Group still produce significantly fewer ΣP-ellipsis utterances than the KA Group, suggesting that the L1 influence concerning the ΣP-ellipsis continues to exist at advanced levels.

### Absence of L1 influence at beginner levels of L2 Chinese oral production

It seems rather unexpected that L1 influence is absent in L1 English and L1 Korean beginners’ L2 Chinese oral production of vP-ellipsis and ΣP-ellipsis, given the Full Transfer (FT) Hypothesis (FTFA, Schwartz and Sprouse, 1994, 1996), which proposes that the initial state of the L2 grammar is entirely based on the final state of learners’ L1 grammar. Assuming that L1 English and L1 Korean beginners in our study are representatives of initial states of L2 Chinese grammars, it would be predicted, on the basis of FT Hypothesis, that beginner learners of L2 Chinese whose L1 disallows a certain target language structure would lag behind those whose L1 has an equivalent of it. However, neither L1 English beginners have any advantage over L1 Korean beginners in their oral production of vP ellipsis, nor L1 Korean beginners have any advantage over L1 English beginners in ΣP-ellipsis, even though vP-ellipsis is allowed in English but disallowed in Koran and ΣP-ellipsis is allowed in Korean but disallowed English. The two groups behave similarly in our study and neither of the groups produce any substantial number of utterances with vP-ellipsis and ΣP-ellipsis in spite of the vP-ellipsis and ΣP-ellipsis in eliciting sentences in the study. These findings do not support the FT Hypothesis. According to this hypothesis, any failure in accommodating target language input will trigger restructuring of the L2 grammar. If L2 initial states are entirely based learners’ L1 grammar, the abundant evidence of ΣP-ellipsis and vP-ellipsis in the Chinese input is expected to trigger changes to learners’ L2 Chinese grammars so that ΣP-ellipsis and vP-ellipsis can be accommodated. However, no change seems to occur in either L1 English or L1 Korean beginners’ L2 Chinese grammars. One may wonder whether the absence of L1 transfer is due to the beginner learners’ difficulty with the basic sentence structures or vocabulary involved in the study. Recall that the participants’ high accuracy in the comprehension task reported in Section “Results of the comprehension test”. Above suggests that learners in all groups have no problem understanding the sentences involved in the study. More importantly, both L1 English and L1 Korean beginner groups produce an overwhelming number of “complete” utterances with no ellipsis in the experiment, which suggests their mastery of the underlying structures involved in the study. These facts indicate that the basic sentence structures and vocabulary involved in the study are available in beginner learners’ L2 Chinese.

Recall that in comparison with utterances with vP- or ΣP-ellipsis, “complete” sentences with no ellipsis are grammatical but stylistically heavy and unconcise in Chinese. It seems likely that no syntax-stylistics interface is established in beginner learners’ L2 Chinese, leading to a breakdown at a syntax-stylistics interface in their handling of sentences with vP- or ΣP-ellipsis. As beginner learners have limited L2 knowledge and unsophisticated coordination between information from different cognitive domains, such as syntax and stylistics, the mechanisms for their L2 oral production tend to be geared for syntactic “completeness” and are unlikely to be susceptible to any stylistic requirement, even though the syntax-stylistics interface is available in their L1s. As a result, this insensitivity to stylistic requirements at L2 initial stages leads to the absence of vP- and ΣP-ellipsis observed in L1 English and L1 Korean beginners’ L2 Chinese oral production. That is, L2 learners’ production at the beginner level is governed exclusively by basic essential syntactic computations and it is immune to stylistic requirements, which overrides L1 transfer of the syntax-stylistics interface from their L1s to their L2 speech production, leading to the absence of the influence of their L1 vP- or ΣP-ellipsis on their L2 Chinese oral production of utterances with vP- and ΣP-ellipsis in the current study.

If the analysis above is on the right track, it is reasonable to assume that, with improved L2 Chinese language proficiency and their increased automaticity in L2 Chinese oral production, and with more exposure to vP- and ΣP-ellipsis in their L2 Chinese input, they are more likely to produce utterances with vP- and ΣP-ellipsis, and this tendency is indeed observed in our intermediate and advanced learners’ data. At the same time, L1 influences are detected at intermediate and advanced levels as well, where English speakers are found to produce more utterances with vP-ellipsis in their L2 Chinese oral production than Korean speakers, and in contrast, Korean speakers produce more utterances with ΣP-ellipsis than English speakers. This finding is on a par with what is reported in Zhang (2020), who focuses on English- and Korean-speaking learners of L2 Chinese at intermediate levels only, which is why the absence of L1 influence is observed in the current study and in Yuan and Zhang (2020), but not in Zhang (2020), where no L2 Chinese speakers at beginner levels are involved. Anyway, we argue that the different behaviours in their L2 Chinese oral production between English and Korean speakers at intermediate and advanced levels are a manifestation of what is allowed and disallowed in their respective L1s. That is, their oral production of these syntactically complicated but stylistically concise utterances is facilitated by the availability of the syntax-stylistic interface in their respective L1s, English and Korean.

The absence of L1 influence at beginner levels found in the present study is also in conformity with the finding concerning beginner learners’ L2 Chinese oral production of utterances with object-ellipsis in Yuan and Zhang (2020), where an incremental model is adapted from Bock and Levelt (1994) for the findings in their study. The model is designed for the planning of speech production, and is assumed to have four stages: (a) lexical selection; (b) functional assignment; (c) constituent assembly; and (d) derivation, which includes checking and deleting. According to this model, the grammatical coding and operations are expected to be implemented before a sentence is phonetically spelt out. On the basis of the incremental model, Yuan and Zhang believe that beginner learners of L2 Chinese in their study have no problem with the first three stages. That is, they are able to select lexical items from their mental lexicon for the meaning to be expressed; they can assign grammatical functions, such as subject, object, etc., to the lexical items selected from their mental lexicon, and they are also able to assemble the lexical items in a word order appropriate to the target language. However, what they are unable to do at beginner levels is implement the derivation, such as checking and deleting, which require additional operations and are therefore more costly and taxing. Although the absence of L1 influence is not specifically addressed in Yuan and Zhang (2020), it seems possible to use their analysis to account for English and Korean beginners’ overwhelming production of L2 Chinese utterance with no vP- and ΣP-ellipsis. We can argue that L2 beginners in our study also encounter problems in dealing with operations at the derivation stage. Production of utterances with vP-ellipsis or ΣP-ellipsis requires verbal identity checking of whether the vP or ΣP in the second sentence is identical to that in the first coordinate sentence before the vP or ΣP in the second sentence is elided. If beginner learners are unable to implement the operations of the required checking and deleting, this would naturally lead to the overproduction of utterances of non-ellipsis and rare production of utterances with vP- and ΣP-ellipsis in their L2 Chinese oral production. Furthermore, the interface issue discussed above is likely to insert an additional layer of complication in the model. That is, as Chinese vP- and ΣP-ellipses operate at a syntax-stylistic interface, L2 Chinese speakers are unable to simultaneously handle, among other things, information from different sources at beginners’ levels, which further reduces their ability to rely on their L1s in their oral production of vP- and ΣP-ellipses in their L2 Chinese oral production.

It should be acknowledged that with the data from and the design of our experiment, we are not in a position to pinpoint whether and to what extent the breakdown at the syntax-stylistics interface or the operations of checking and deleting is the main reason behind the absences of L1 transfer in beginner learners’ L2 Chinese oral production of utterances with vP- and ΣP-ellipsis. It is likely to be a joint effect of the two. We have to leave this issue for future research.

#### Asymmetry in disappearance of L1 influence

Another interesting finding in the current study is that L1 influence disappears earlier in L2 Chinese production of vP-ellipsis than ΣP-ellipsis. The two advanced groups perform similarly in their Chinese oral production of vP-ellipsis, but the Korean group seems to continue to have the advantage of the ΣP-ellipsis in their L1 Korean and produce significantly more L2 Chinese utterances with ΣP-ellipsis than English speakers. In order to explain the asymmetry, it seems necessary to take into account differences in structural complexity between vP-ellipsis and ΣP-ellipsis. As discussed in “Cross-linguistic differences of verb-phrase ellipsis in Chinese, Korean and English”, in comparison to a vP, a ΣP involves an ellipsis of a bigger constituent and its scope can include a negator, a model verb as well as a vP, as shown in Chinese sentences like (3) in “Cross-linguistic differences of verb-phrase ellipsis in Chinese, Korean and English”. We believe that what is elided can be measured on the basis of computational complexity involved, which, in turn, can affect early or late disappearance of L1 influence. The asymmetry in the disappearance of L1 influence in speech production of vP- and ΣP-ellipsis at advanced levels can be accounted for with the help of the measurement of computation complexity in feature checking, as in Yuan (2015), who proposes that “Feature checking of α gives rise to a less complex computation than feature checking of α*+* β” (Yuan, 2015, p.8). We can adapt his proposal and apply it to the analysis of the late disappearance of L1 influence on ΣP-ellipsis in English speakers’ L2 Chinese, by assuming that verb identity checking of α alone gives rise to a less complex computation than verb identity checking of α+β and that deleting only α gives rise to a less complex computation than deleting α+β.

According to this metric, the more items a verbal identity checking operation involves, the more computational complexity the operation has. L2 structures with less computational complexity are expected to be acquired more easily than those with more computational complexity. In the current case, the operations on the vP-ellipsis are computationally less complex than the ΣP-ellipsis, because the former involves identity checking and deleting of only a vP and an NP object, but the latter requires identity checking and deleting of not only a vP and an NP object, but also a model and a negator. Although Korean does not have the vP-ellipsis, L1 Korean learners are able to overcome the disadvantage of not having vP-ellipsis in their L1 and acquire the less complex vP-ellipsis construction at a relatively early stage in their L2 Chinese acquisition. In contrast, L1 English learners do not allow the ΣP-ellipsis in their L1. In addition, the ΣP-ellipsis in the target language Chinese is computationally more complex than the vP-ellipsis, requiring identity checking and deleting of more items than vP-ellipsis, and as a result, their L1 English grammar is relied upon more when Chinese sentences with ΣP-ellipsis is dealt with, delaying the disappearance of L1 influence in their L2 Chinese oral production of utterances with ΣP-ellipsis. This explains why L1 influence is shorter-lived in L2 Chinese oral production of vP-ellipsis than ΣP-ellipsis.^6^

## Conclusion

The current study tracks the role of L1 in L2 speech production of Chinese verb phrase-ellipsis structures at different stages of L2 development. One finding of the study is the absence of L1 influence on L2 Chinese speech production until intermediate and advanced levels. Both L1 English and L1 Korean learners of L2 Chinese at beginner levels tend to produce complete responses with no ellipsis, in spite of the fact that vP-ellipsis is allowed in English and that ΣP-ellipsis in Korean. This finding provides us with evidence against the FT Hypothesis proposed by (FTFA, Schwartz and Sprouse, 1994, 1996), which proposes that the initial state of the L2 grammar is entirely based on the final state of learners’ L1 grammar. At intermediate levels, English- and Korean-speaking learners produce significantly more utterances with the type of ellipsis allowed in their L1s. The different behaviours between L2 learners at beginner and intermediate levels are attributed to a breakdown at the syntax-stylistics interface and to the difficulty caused by the identity checking and deleting operations involved in the derivation stage in beginner learners’ L2 Chinese speech production. L2 learners at beginner levels are believed to strive for syntactic completeness and derivational simplicity before implementing syntactic approaches to stylistic modification, which overrides L1 transfer in beginners’ L2 Chinese speech production. Another finding is the difference in the persistence of L1 influence on the two types of ellipsis in English and Korean speakers’ L2 Chinese oral production; with regard to vP-ellipsis, L1 influence can be caught at intermediate levels but disappears at advanced levels; with regard to ΣP-ellipsis, L1 influence seems to be longer-lived, as it continues to exist at advanced levels. This is accounted for on the basis of a modified version of the computational complexity metric in Yuan (2015). Based on the finding in the current study, we argue that L1 influence should be considered a relative phenomenon in L2 speech production, and its presence and absence can be related to a number of factors, including learners’ ability in handling information from different cognitive domains at interface levels, the availability of operations at derivation stages in their L2, the computational complexity involved, etc. Of course, it deserves further research as to which of these factors plays a more important or decisive role in L1 influence in L2 oral production.

## Data availability statement

The original contributions presented in the study are included in the article/Supplementary material, further inquiries can be directed to the corresponding author.

## Ethics statement

Ethical review and approval was not required for the study on human participants in accordance with the local legislation and institutional requirements. The participants provided their written informed consent to participate in this study.

## Author contributions

LZ did the data collection and statistical analyses. BY made substantial contributions to the theoretical framework for the findings of the study. LZ and BY made equal contributions to the design of the experiment, data interpretation, and the writing of the manuscript. Both authors contributed to the article and approved the submitted version.

## Funding

The work was supported by Shanghai Planning Office of Philosophy and Social Science (Grant No. 2022EYY009).

## Conflict of interest

The authors declare that the research was conducted in the absence of any commercial or financial relationships that could be construed as a potential conflict of interest.

## Publisher’s note

All claims expressed in this article are solely those of the authors and do not necessarily represent those of their affiliated organizations, or those of the publisher, the editors and the reviewers. Any product that may be evaluated in this article, or claim that may be made by its manufacturer, is not guaranteed or endorsed by the publisher.

## Footnotes

^1^The full name of the model proposed by Schwartz and Sprouse (1994, 1996) is Full Transfer and Full Access Hypothesis. As we are only concerned with the transfer part, but not the access part, of the model in this article, we, hereafter, simply call the model Full Transfer (FT) Hypothesis to highlight our focus on the transfer part of the model.

^2^The term *stray affix* in Lasnik (1981)is used to describe a situation where a verbal suffix is illicitly used independently in languages like Japanese and Korean.

^3^As pointed out by the handling editor, unlike *shi 是* ‘BE’ in Chinese, which is a free morpheme, *ya*야 in Korean is a bound morpheme which has to be attached to a verbal element. What is in common between the two, though, is that both of them can license a ΣP-ellipsis in their respective languages. Also, as mentioned by a Korean native speaker, both *ya*야 and *yo*요 can license a ΣP-ellipsis. *Ya* is used in informal sentences while *yo* is a formal form. The current article follows Kim and Sohn (1998) and only uses *ya* in Korean examples.

^4^The elicited imitation task is called *utterance-recall task* in Yuan and Zhang (2020).

^5^An acceptability judgement task and a picture-matching judgment task were also conducted, but the results of these two tasks are not to be reported here as they are irrelevant to the research questions discussed in the current paper.

^6^This analysis seems to tie in well with the finding in Yuan and Zhang (2020) that no L1 influence is observed in L2 Chinese production of object-ellipsis by L1 English and L1 Korean learners. Identity checking and deleting of just the NP object is computationally even less complex than vP- and ΣP-ellipsis. Consequently, the L1 influence involved is expected to disappear even earlier. The reason that no L1 influence is observed in L2 Chinese production of object-ellipsis is probably because the L1 influence involved is too short-lived to be caught in Yuan and Zhang’s (2020) study.
